# Neutering Effects on Social Behaviour of Urban Unowned Free-Roaming Domestic Cats

**DOI:** 10.3390/ani9121105

**Published:** 2019-12-09

**Authors:** Simona Cafazzo, Roberto Bonanni, Eugenia Natoli

**Affiliations:** 1 Independent researcher, Via Giovanni Cagliero 17, 20125 Milan, Italy; simona.cafazzo@gmail.com; 2 Independent Researcher, via Giuseppe Donati 32, 00159 Rome, Italy; rbonanni75@gmail.com; 3 Canile Sovrazonale, ASL Roma 3, Via della Magliana 856H, 00148 Roma, Italy

**Keywords:** unowned domestic cats, neutering, social behaviour, activity level

## Abstract

**Simple Summary:**

The “trap, spay/neuter, and release” programs to manage unowned free-roaming cat populations are diffused worldwide and they are largely advised even in countries where the suppression of unowned cats is enforced by law. Despite the massive neutering campaigns in the world, there is little information on the influence of neutering on individual cat behaviour, as well as on the social structure of cat colonies. The aim of this study is to investigate such effects. Before neutering, the group consisted of 17 free-roaming domestic cats, who were totally unrestrained. After neutering it consisted of 16 individuals. The social behaviour of cats, living in the group, was registered, using classic ethological methods (735 h of observation before and 537 h after neutering). The social structure did not change after neutering, but cats became less active. The results of this study suggest that, after neutering: (i) The group of free-ranging cats was stable over time; (ii) the hierarchical structure of the cat social group did not change; (iii) the general level of activity, decreased; (iv) urine spraying marking behaviour almost disappeared; (v) the level of proximity decreased although it tended to increase in a few male–male dyads.

**Abstract:**

The “trap, spay/neuter, and release” programs to manage unowned free-roaming cat populations are diffused worldwide and they are largely advised even in countries where the suppression of unowned cats is enforced by law. Despite the massive neutering campaigns in the world, there is little information on the influence of neutering on individual cat behaviour, as well as on the social structure of cat colonies. The aim of this study is to verify such effects. Before neutering, the group consisted of 17 free-roaming domestic cats, who were totally unrestrained. After neutering it consisted of 16 individuals. Data on the outcomes of aggressive, submissive, affiliative, and territorial behaviour were collected, using classic ethological methods (“focal animal”, “all occurrences”, and “1/0” sampling methods) (735 h of observation before and 537 h after neutering). The dominance hierarchy did not change after neutering. On the other hand, the frequency of aggressive, territorial behaviour as well as proximity among individuals decreased significantly. Affiliative behaviour has been observed among neutered adult males that never performed it before neutering. The results of this study suggest that, after neutering: (i) The group of free-ranging cats was stable over time; (ii) the hierarchical structure of the cat social group did not change; (iii) the general level of activity, decreased; (iv) urine spraying marking behaviour almost disappeared; (v) the level of social proximity among group members decreased, although it tended to increase in some male–male dyads. The most obvious effect of neutering, detected on individual behaviour, was that cats were less active.

## 1. Introduction

In Italy, the control of the demography of unowned free-roaming cats (All domestic cats without an owner who have no constraints on their movements and on their breeding (reviewed in [1,2])), living in colonies in the urban environment, is regulated by the national law no. 281, enacted in 1991. Furthermore, each Italian region has to have a regional law that has implemented the national one. For example, in the Lazio region, apart from the first law on the control of unowned free roaming dogs and cats (no. 63 come out in 1988) that was promulgated before the national one, the second law implemented the 1991 national law (no. 34 come out in 1997).

The National law cited, which has been the first “no-kill policy” law in the world, implemented the “trap, spay/neuter, and release” (TNR) programs for unowned domestic cats [1] and represented a “revolution” for the management of these animals; in fact, among the most important points the law established that: (i) Urban free-ranging cats have the right to live free; (ii) they have to be neutered by the local Public Veterinary Services and reintroduced in their colony; (iii) urban free-ranging cats cannot be abused or moved from their colony; and (iv) “free-ranging/stray cats cannot be euthanized unless they are incurable or proven to be dangerous”.

After the promulgation of these laws, the Veterinarian Public Services (VPS) have assumed a crucial role in the demographic control of urban free-ranging cat colonies. Cat colonies living within the Municipality of Rome are registered by VPS since 1989 on the basis of (a) citizen requests for neutering or (b) the signaling of hygienic and/or sanitary problems concerning the presence of cats. After registration, colonies are censused by the VPS; cat lovers are responsible for capturing the cats who are then surgically neutered by the VPS and reintroduced in their original colony.

So far, in our country, despite the massive sterilization campaigns, there are few published papers on the results obtained, from a quantitative (see for example [2,3]) but especially from a qualitatively point of view, i.e., on the influence of neutering on either cat behaviour or on the social structure of cat colonies. In other words, despite the recent worldwide considerable examination in investigating and discussing TNR programs, the researches rarely focused on changes due to neutering in cat social relationships and in the resulting social structure of the cat group.

The available literature is mainly focused on the effects of TNR programs in unowned free-roaming cats, i.e., on their effectiveness based on the time of application (reviewed in [4], but see also [1]) and on the effects of neutering on the extent of the home range of cats [5]. Some works have as their object the influence of neutering on the welfare of cats [6,7] or on the dangers to their health [8], or on the age of which it is correct/ethical to practice neutering [9]. But very few studies, and for the most part dated, have analyzed if and how the social relationships of cats living in groups change following the neutering of all cats in the group (see for example [10,11]).

The aim of this study is to verify the effects of neutering on social relationships and on the social structure of a group of free-roaming cats without owners, living in the urban environment.

## 2. Materials and Methods

### 2.1. Study Area

The study was carried out in a courtyard located in an urban sector of Rome (Italy). Despite the fact that the cat colony lived in such an environment, the large courtyard (about 6000 m^2^) was bounded by a wall and partly isolated from road traffic. The courtyard contained 11 buildings, open areas, flower-beds cultivated by inhabitants, trees, and bushes. Cats were free to wander in every part of the courtyard, and they had access to a regular food supply provided by the people living around the courtyard every day, putting it regularly in a feeding site placed in a small field (about 150 m^2^). Adjacent to it, cats established their ‘core area’ in a partially wire-fenced sector located in the south side of the courtyard. Here, a lot of spontaneous vegetation offered good shelter for animals, especially for lactating females with kittens. Cats were rarely seen hunting. Water was available from a fountain just outside the entrance of the courtyard.

### 2.2. Description of the Cat Group

The group consisted of 15 unowned free-roaming domestic cats, plus 2 owned individuals who were totally unrestrained and interacted with the unowned cats on a regular basis.

Before the commencement of the study it was noted that 2 females in the colony were already neutered. The unneutered population consisted of 3 entire adult females, 8 entire adult males, 2 entire male kittens, and 2 entire female kittens. During the first period of study, before neutering, 2 adult males dispersed and 7 kittens were born from females belonging to the group studied. Therefore, after neutering the colony consisted of 16 individuals, 9 of which were present before neutering (5 neutered adult females, 4 neutered adult males) plus the 7 entire kittens. During the period of the study the number of cats decreased to 10 individuals as a consequence of an infectious viral bronchopneumonia. After neutering, the statistical analysis was performed on 10 individuals (4 adult males, 5 adult females, and 1 juvenile male) in the absence of any sources of competition and on 14 individuals (3 adult males, 4 adult females, 3 juvenile males, and 4 juvenile females) in the presence of food (see Table 1 for more details). The higher number of individuals tested in presence of food was due to the fact that juveniles were involved in the competition for food, while never participated in agonistic encounters with adult cats.

All individuals were individually recognized by their coat color pattern, hair length, eye shape and color, bushiness of tail, size and scars on the ears. The sex of adult cats could be determined on the basis of morphological (presence of testes, wider face and larger body size in males) and behavioural characteristics (posterior urine spraying of males, rarely displayed by females).

They had no constraints placed or movements by human owners and very few individuals were tame enough to be handled.

Age of the subjects was obtained from cat care-takers who fed the cats, or estimated according to the method described in [12]. Cats were considered to be kittens up to the age of 6 months, to be juveniles from 7 to 11 months, to be sub-adults from 12 to 18 months and to be adults afterwards [13].

### 2.3. Experimental Procedure

#### 2.3.1. Data Collection

Between September 2001 and June 2002, when the majority of the group members were not neutered, a study was carried out in order to verify the existence of a dominance hierarchy among the cats belonging to the group, as well as the influence of the rank, sex and age on the order of access to food [14]. Subsequently, from July to September 2002, some members of the group were captured using trapping cages containing food for cats; other cats of the group were captured after being anesthetized using a syringe containing ketamine, shot from a blowgun. The cats were transferred to the public dog shelter of Rome where all colony cats are neutered by law by the vets belonging to the Public Veterinary Services, before being released in their original colony.

Trapped individuals were anaesthetized with ketamine chlorohydrate (Inoketam 1000, 5 mg/kg, Virbac, Milan, Italy) and medetomidine (Domitor, 0.005 mg/kg, Pfizer, Rome, Italy).

Neutering was performed through a surgical operation to remove the gonads: The ovarectomy for the females and the orchiectomy for the males; immediately after the operation each individual was marked with the removal of the tip of the right ear, as required by the Italian laws. The neutered animals, after a 3-day permanence at the sanitary unit of the dog shelter, were released in the study area.

From October 2002 to June 2003, 428 h of observation were carried out in the absence of food and 109 h in presence of food; a total of 537 h of observation were carried out by mean of ‘Focal animal sampling’ method [15], in order to collect data on the outcomes of aggressive, submissive as well as affiliative and territorial interactions of the cats. Moreover, the ‘Focal subgroups sampling’ method [15] was utilized in order to collect data around the food (for a total of 100 sessions). The same procedures for collecting behavioural data were utilized before and after neutering [14].

In the absence of any sources of competition the individual observation time was distributed all day long, between 8:00 a.m. and 8:00 p.m., and attempts were made to distribute it evenly among the members of the group. The behavioural patterns in order to code for each behavioural system outcomes (aggressive, affiliative etc.) were chosen on the basis of a combination of the dictates of historical studies on the social behaviour of mammals (see for example [16]) matched with our consolidated experience in behavioural studies on colony cats in the urban environment (see for example [17]). Agonistic behaviour (composed by aggressive and submissive behaviour) in the absence of food was recorded by ‘All occurrences’ method [15]. Aggressive behaviour included the following: Threats (striking with a paw, biting, assuming threatening postures, pointing, staring at, baring of the canines), chasing, ritualized vocal duels, and real duels. Submissive behaviour included: Crouching with the ears flattened, avoiding, retreating, fleeing, and hissing at. Kittens were not included in this data collection because they very rarely interact agonistically with adults.

During the observation sessions, we also recorded all territorial (urine marking and rubbing the perioral region on objects) and some affiliative (sniffing nose, body rubbing) behaviours displayed by cats by using the all occurrences method [15]. Other two affiliative behaviours, passive contacts and allogrooming, were recorded by using the “One-zero” method [15]. The individual scores of all behavioural patterns were corrected for animal observation time because, despite the attempts to distribute it evenly among the members of the group, cats entered and went out of sight when they decided; as a consequence, the individual observation time sometimes varied between individuals.

Finally, during focal sessions we also used the ‘Instantaneous sampling’ method to collect data on the time that cats spent within 1 m of distance from each other.

Food distribution times were set at 9:30 a.m. and 2:30 p.m., respectively. Each observational session began as soon as the person usually responsible for feeding the cats placed the food bowl on the ground. The food consisted of about 400 g of canned meat for cats. The duration of each observation session was generally an hour; however, on occasions when cats took longer to consume all the food available, the experiment was extended further until the food was completely exhausted. In order to assess the dominance hierarchy in the presence of food, we utilized the ‘all occurrences’ method for recording all agonistic interactions among cats within 1 m from food. These included the aggressive and submissive behavioural patterns described above, plus ‘interruption of feeding after receiving an aggression’ that was a submissive behaviour. Individual scores were corrected for presence scores around the food.

#### 2.3.2. Data Analyses

In order to determine the dominance hierarchy in both contexts analyzed (in the absence of any sources of competition and in the presence of food), the distributions of dyadic aggressive and submissive interactions were ranked in two different squared matrices with performers on one axis and recipients on the other, so as to minimize the number of dominance reversals. The dominant animal of each dyad was the one who performed more aggressions than he/she received, or received more submissions than he/she performed. Hissing was excluded from the matrix based on submissive interactions because it was considered an ambiguous behaviour, with components both of aggressiveness and subordination. We tested the transitivity of dominance relationships between the members of the social group, based either on aggressive or on submissive behavioural patterns separately, by applying an improved test of linearity developed by [18], which is based on Landau’s linearity index but takes into account unknown and tied relationships between group members. Then we determined the dominance rank order by using the I&SI method which minimizes inconsistencies and strengths of inconsistencies in dominance relationships [18]. An inconsistency occurs when individual j dominates i, and j’s rank is lower than i’s [8]. The rank difference between two individuals involved in an inconsistency is the strength of that inconsistency [18].

Dominance hierarchy was determined on 10 individuals in the absence of any sources of competition and on 14 cats in the presence of food. The discrepancy in the number of animals included in the analyses was due to the death of some of them during the period of the study and to the absence of two of them during feeding. In fact, in the absence of sources of competitions we excluded from the analysis 6 juveniles (TAR, MAC, CIC, STE, WHI, and NAN) which died after four months from the beginning of the second period of the study and for which we did not have enough data for including them in the hierarchy. In the presence of food, it was not necessary to exclude the 6 juveniles since they participated to enough feeding session to be included in the analysis; however, we had to exclude 2 adults (PIC and RIG) since they never participated to feeding sessions.

To investigate the possible variation of dominance relationships between group members, we correlated the rank order after neutering to the one before neutering by including in this analysis only cats presents in both period on the study for which we collected behavioural data in both periods (7 individuals: 3 adult males and 4 adult females). Practically, we had to exclude from this analysis 2 cats, SPO and RUG, which were juveniles before neutering and for which we did not have data in such period.

For all the analyses concerning the comparison of behavioural frequencies we considered only those cats which were entire before neutering and all cats for which we had data in both period. Therefore, all the following analyses included only 6 of the 7 adult individuals for which data were available before and after neutering, since one of them, PIC, was already neutered during the first period of the study.

In order to analyze the variation in the time that cats spent close to each other, we calculated the Dyadic Association Indexes (DAIs) for each dyad before and after neutering, following [19].

To investigate the variation in the frequency of all behavioural patterns recorded before and after neutering as well as on the time cats spent close to each other, we applied the Wilcoxon signed rank test. From these analyses we excluded one cat, PIC, who was already neutered during the first phase of the study.

To analyze data at the dyadic level, we used Matman’s Row-wise matrix correlation tool with 1 × 104 permutations (MatMan 1.0 Software, Noldus^®^ Information Technology, Wageningen, The Netherlands) to check for a possible correlation of dyad association indexes with frequencies of affiliative behavioural patterns.

Linearity and I&SI rank orders were calculated using Matman 1.1 (10,000 randomizations; Noldus Information Technology, Wageningen, The Netherlands) [20]. Spearman’s rank correlations and Wilcoxon tests were calculated in STATISTICA 7.1 edition (StatSoft Italia srl, 2005, Vigonza, Italy).

## 3. Results

A linear dominance hierarchy based on the outcome of submissive behaviour was found before neutering both in the absence of any sources of competition (linearity index h′ = 0.45 *p* = 0.03, [14]) and in the presence of food (h′ = 0.46 *p* = 0.009, [14]). After neutering we found again a dominance hierarchy in both context, even more linear than the one before (in the absence of any sources of competition: h′ = 0.62, *p* = 0.03; in the presence of food: h′ = 0.53, *p* = 0.002) (Table 2 and Table 3). The rank order found in the absence of any sources of competition was highly correlated to that established before neutering (rs = 0.96, *n* = 7, *p* = 0.0005; Table 4, Table S1), while the one found in the presence of food was exactly the same established before neutering: rs = 1, *n* = 7; Table 5, Table S2).

**Figure 1:**
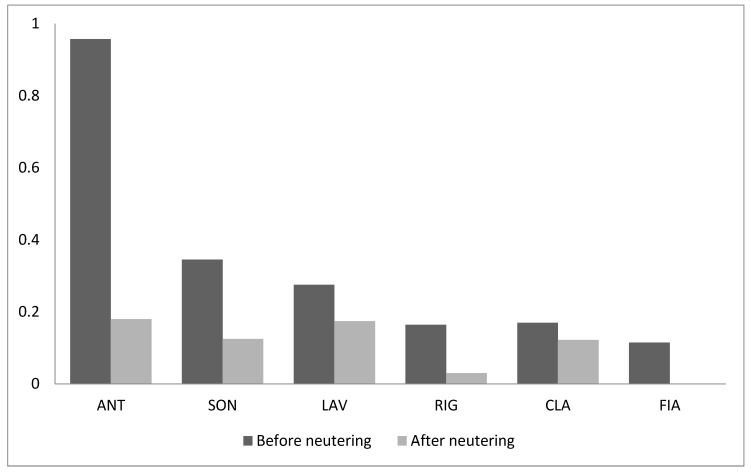
Frequency of aggressive behaviour (acts/h) in the absence of any source of competition before and after neutering.

**Figure 2:**
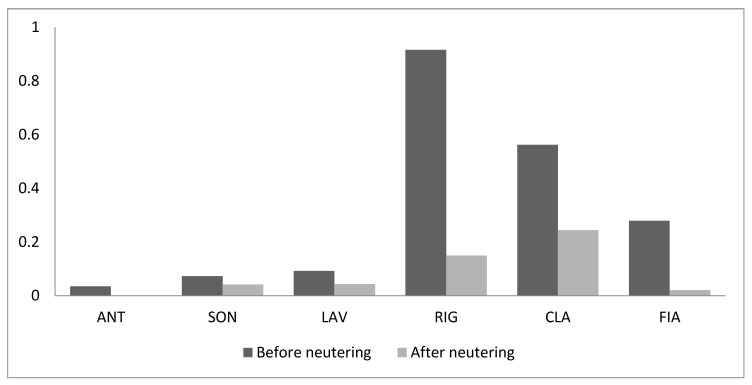
Frequency of submissive behaviour (acts/h) in the absence of any source of competition before and after neutering.

Although a dominance hierarchy clearly still exist among cats despite of neutering, in the absence of any sources of competition the frequency of both submissive and aggressive behaviour decreased significantly (Wilcoxon test: T = 0, *n* = 6, *p* = 0.027, both behavioural categories) (Figure 1 and Figure 2).

**Figure 3:**
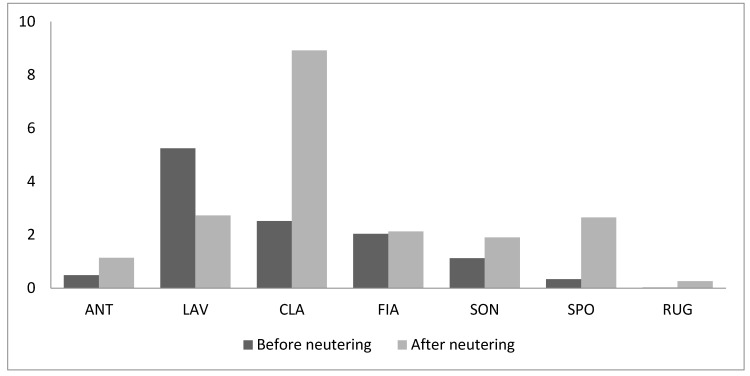
Frequency of aggressive behaviour (acts/h) in the presence of food before and after neutering.

**Figure 4:**
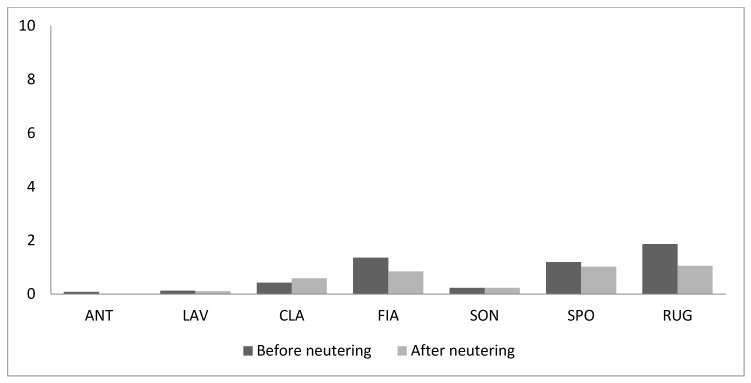
Frequency of submissive behaviour (acts/h) in the presence of food before and after neutering.

On the contrary, we did not find any significant variation in the frequency of both submissive (T = 4, *n* = 7, *p* = 0.09) and aggressive behaviour (T = 6, *n* = 7, *p* = 0.18) in the feeding context (Figure 3 and Figure 4).

**Figure 5:**
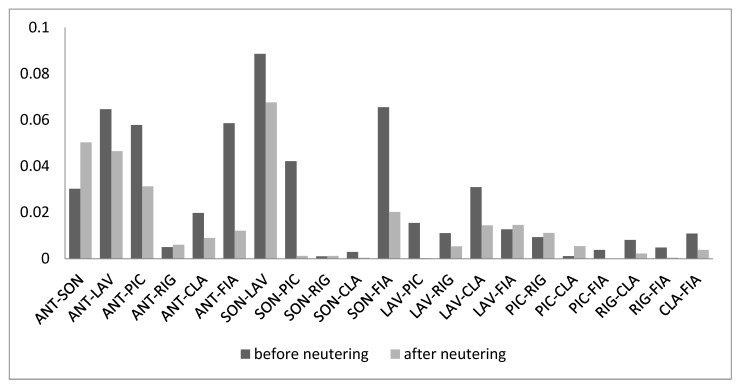
Dyadic association index before and after neutering.

After neutering cats spent less time close to each other within 1 m (T = 33, *n* = 21, *p* = 0.004; Figure 5). Nevertheless, the time they spent close to each other between 1 and 4 m did not change (T = 76, *n* = 21, *p* = 0.17).

Overall, we did not find any significant variation in the frequency of affiliative behavioural patterns.

**Figure 6:**
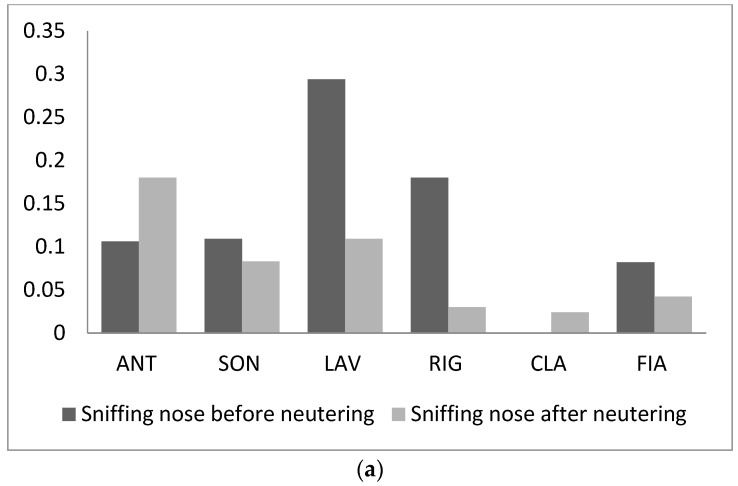

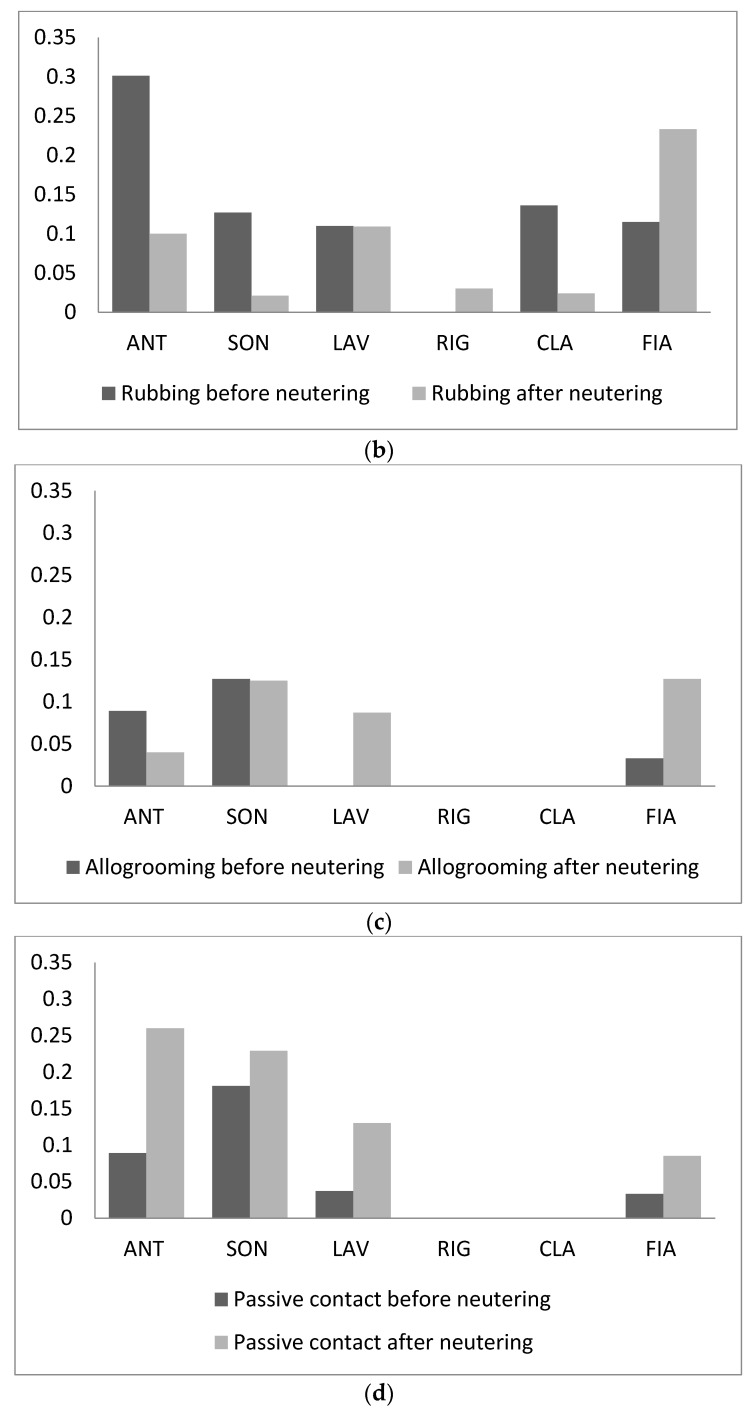
Frequency of all affiliative behavioural patterns (acts/h) recorded before and after neutering: (**a**) sniffing nose, (**b**) rubbing, (**c**) allogrooming, and (**d**) passive contact.

In particular, sniffing nose and rubbing increased in frequency only for two individuals (sniffing nose: ANT and CLA; rubbing: RIG and FIA) and decreased for all other cats (Figure 6a,b); overall, their variation was not significant (sniffing nose: T = 5, *n* = 6, *p* = 0.25; rubbing: T = 7, *n* = 6, *p* = 0.46). It is worth noting that rubbing and sniffing nose have been observed among adult males that never exchanged it before neutering (ANT-SON and ANT-RIG). The same males spent also slightly more time close to each other than before sterilization. Indeed, both behavioural patterns were positively correlated with the dyadic association index (Row-wise matrix correlation: Sniffing nose, Kr = 19, τrw = 0.60, *n* = 6, *p* = 0.005; rubbing; Kr = 18, τrw = 0.56, *n* = 6, *p* = 0.007).

Allogroming and passive contact were not statistically analyzed since they were performed only by few cats in the colony. For the three cats that performed allogrooming (Figure 6c) before and after neutering, the frequency decreased slightly; one cat started showing this behaviour only after neutering. Finally, passive contact increased in frequency in all four cats that showed it before and after neutering (Figure 6d).

**Figure 7:**
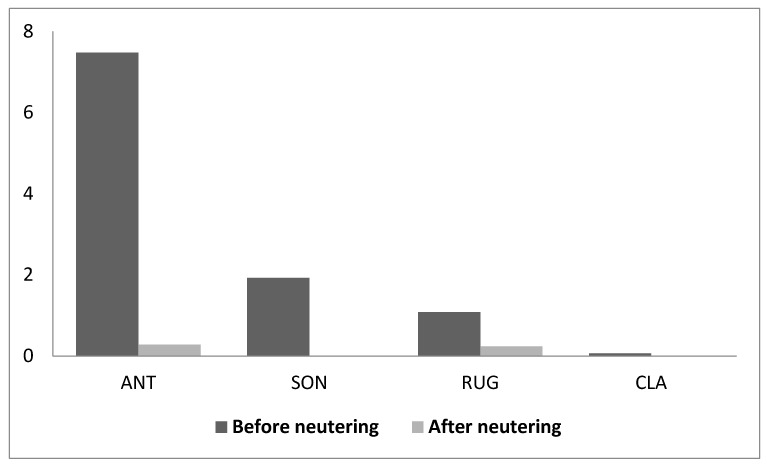
Frequency of marking behaviour by means of sprayed urine (acts/hour) before and after neutering.

Urine marking decreased or disappeared completely in all cats which displayed it before neutering. However, since only 4 cats (3 adult males and one adult females) displayed this behaviour, the statistical analysis failed to rich the significant level (T = 0, *n* = 6, *p* = 0.06) (Figure 7).

**Figure 8:**
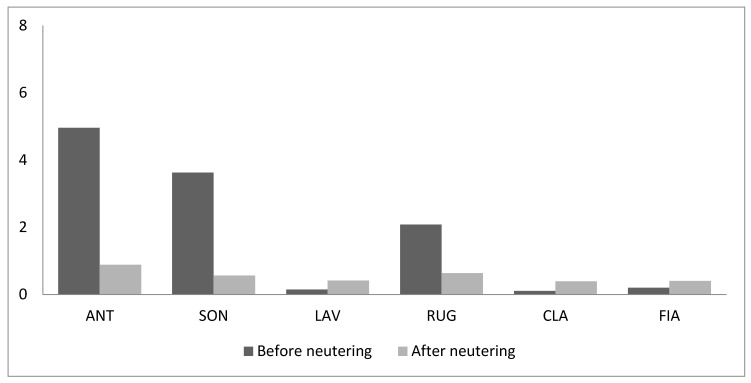
Frequency of marking behaviour by means of rubbing the perioral region on objects (acts/hour) before and after neutering.

On the contrary, the frequency of rubbing the perioral region on objects did not change (T = 6, *n* = 6, *p* = 0.35) (Figure 8).

It is interesting to note that a lot of behaviour change occurred in 4 months after neutering because in September 2002 all cats of the group were neutered and the second part of the study (data collection) lasted from September 2002 to June 2003.

## 4. Discussion

One of the most relevant result of this research is the finding of a linear dominance hierarchy among the members of the cat colony, despite all of them had been neutered. This result support the findings of valid but not recent study on free roaming urban cats that had led to the same results [10]. Furthermore, after neutering the hierarchical order of the animals did not change, in the absence of resources and in presence of food. It is possible that the hierarchy remained stable over time because neutering reduced the incentives to challenge those who are on top of the hierarchy.

The acquisition of this information on the behaviour and organization of urban domestic cats is fundamental for their management in the perspective of respecting their well-being. For example, it is well known that the reorganization of social relationships is an important cause of stress in many mammalian species (see for example [21,22]). Although the instability of the hierarchy is a temporary phase and it is not said that the acute stress becomes chronic for all members of a social group (but it can occur for some of them [21]), it is better to avoid a human intervention invasive such as neutering only for some members of a social group of cats. Since dominance relationships among all members of our cat colony did not change after neutering, maybe neutering ALL members of the group might mitigate the social stress in cats caused by the reorganization of social relationships [7] provoked by the neutering of only some individuals.

Our results show that neutering had a significant effect on aggressive behaviour, because it decreased substantially when no resources were present. Being a testosterone-dependent behaviour [23], for males this behavioural response is predictable, and the contextual decrease in submissive behaviour might be related. It is more difficult to explain why neutering had the same effect on females. In general, it is possible that all cats simply became less active. But in front of food, the aggressive and submissive behaviour maintained their importance to gain the resource, and then neutering had no effect.

Despite the fact that the frequency of some affiliative behavioural patterns that cats exchanged was not influenced by neutering, we found that neutering was associated to a statistically significant decrease in the time spent by group members in close proximity to each other (within 1 m). This change might have been due to hormonal changes, such as the fall in testosterone levels in males which, in turn, might have caused a decrease in the male motivation to maintain proximity relationships with females. Moreover, a decrease in testosterone levels and, consequently, in the frequency of aggressive behaviour, can also explain why some affiliative behavioural patterns (rubbing and sniffing nose) were observed in interactions between some adult males only after neutering. The same males involved in these affiliative interactions also spent a little more time next to each other than before neutering. Actually, we found a correlation between the frequencies of two affiliative patterns, i.e., rubbing and sniffing nose, and time spent close to each other. Unfortunately, literature on effect of castration on male social behaviour is lacking, and one paper available is dated [11]; the literature is focused more on neutering effects on cat ranging behaviour, and it reports contrasting results: On one hand, little evidence was found that neutering markedly reduces ranging behaviour for either sex, at least for cats neutered as adults [5], and on the other hand caretakers believed (qualitative observation) that neutering was associated with behaviour changes that included decreased roaming, fighting, and increased friendliness [6].

Finally, it is not surprising that urine marking decreased or disappeared completely in male cats which displayed it before neutering since, again, it is a testosterone-dependent behaviour [23]. Since it is well known that for the domestic cat the territorial marking behaviour by means of urine spraying is the most important way to mark the territory [24], neutering might drastically reduce the capacity of domestic cats to protect their territory from trespassing conspecifics [25]. Thus, for management purposes, the crucial question is: Is the geographical area occupied by a colony of neutered unowned free-roaming domestic cats unavailable to conspecifics? Actually, available data seem to confirm that neutering alters cat territorial behaviour [24,25] allowing immigration from other groups. Much less known in cats are the cause and the function of rubbing the perioral region on objects, on which neutering had no effect.

In summary, the results of this study suggest that, after neutering: (i) The hierarchical structure of the cat social group did not change; (ii) the general level of activity, decreased; (iii) urine spraying marking behaviour almost disappeared; (iv) the level of close spatial proximity among group members decreased, although it tended to increase in a few male–male dyads. The most obvious effect of neutering, detected on individual behaviour, is that cats are less active and show less agonistic behaviour with each other, and that behaviour change occurred in a very short time after neutering.

## 5. Conclusions

This kind of study is very important to improve the management of unowned free-roaming domestic cats in the urban environment, considering that they are diffused all over the world and that the efforts to demographically control them is increasing; the most diffused approach is trap, neuter, and release (TNR) [1]. The influence of neutering on several aspects of domestic cat social behaviour is still not enquired sufficiently. This study supports the view that neutering unowned cats living in urban colonies contributes to decreasing the bad smell due to male cats’ sprayed urine, the disturbance due to vocal duels of competing males and due to vocalizations of females in estrus; consequently, it contributes to decrease the complains of citizens for them; tomcats are less exposed to risk of being infected by feline immunodeficiency virus (FIV) [26]. Future studies should aim at clarifying the influence of neutering on cat social behaviour, because they are crucial for management decision making.

## Figures and Tables

**Table 1:** Variation in the group composition during the periods of the study.

Name	Sex	Periods of Study	
		Before Neutering (Sep. 2001–Jun. 2002)	After Neutering (Oct. 2002–Jun. 2003)
Leoncino (LEO)	Male	Present, adult	Dispersed, adult
Antonio (ANT) *°	Male	Present, adult	Present, adult
Sonny (SON) *°	Male	Present, adult	Present, adult
Pallino (PAL)	Male	Present, adult	Died, adult
Pelliccione (PEL)	Male	Present, adult	Dispersed
Righetto (RIG) *	Male	Present, adult	Present, adult
Gnocco (GNO)	Male	Present, adult	Died
Red (RED)	Male	Present, adult	Died
Lavinia (LAV) *°	Female	Present, adult	Present, adult
Piccoletta ^#^ (PIC) *	Female	Present, adult	Present, adult
Claudia (CLA) *°	Female	Present, adult	Present, adult
Fiaschetta (FIA) *°	Female	Present, adult	Present, adult
Silvestrina (SIL)	Female	Present, adult	Died
Calicò ^a^ (CAL)	Female	Present, kitten	Died
Ruggine ^a^ (RUG) *°	Female	Present, kitten	Present, adult
Spotted ^a^ (SPO) *°	Male	Present, kitten	Present, adult
Nero ^a^ (NER)	Male	Present, kitten	Died
Tartaruga ^a^ (TAR) °	Female	Newborn	Present, juveniles
Macchiola ^a^ (MAC) °	Female	Newborn	Present, juveniles
Cicì ^b^ (CIC) °	Male	Newborn	Present, juveniles
Stefy ^b^ (STE) °	Male	Newborn	Present, juveniles
Fly ^b^ (FLY) *°	Male	Newborn	Present, juveniles
White ^b^ (WHI) °	Male	Newborn	Present, juveniles
Nanà ^c^ (NAN) °	Female	Newborn	Present, juveniles
Total		17	16

Totals are referred to the number of cats present at the beginning of each study period. Individuals are listed in the age order from older to younger. Sep. = September; Jun. = June; Oct. = October. ^#^ She was already neutered in 2001. ^a^ Lavinia’s offspring. ^b^ Claudia’s offspring. ^c^ Fiaschetta’s offspring. * Individuals included in the final statistical analyses after neutering in the absence of sources of competition. ° Individuals included in the final statistical analyses after neutering in the presence of food of competition.

**Table 2:** Dominance hierarchy based on submissive interactions (except hissing) after neutering, in the absence of any sources of competition. Individuals are listed in the rank order. Grey background: diagonal dividing line.

	ANT	LAV	SON	PIC	RIG	SPO	CLA	FIA	RUG	FLY	Tot
**ANT**											0
**LAV**	4										2
**SON**	2										4
**PIC**	2	1									3
**RIG**	8		1	1							10
**SPO**	5	2	1						1		9
**CLA**	4	2		2	1						7
**FIA**	1	3					3				9
**RUG**		1	2		1	2					6
**FLY**	1	1	1		1	1		4	1		10
**Tot**	27	5	10	3	3	3	4	3	2	0	60

**Table 3:** Dominance hierarchy based on submissive interactions (except hissing) after neutering, in the presence of food. Individuals are listed in the rank order. Grey background: diagonal dividing line.

	ANT	LAV	CLA	FIA	SON	SPO	TAR	FLY	CIC	RUG	MAC	STE	WHI	NAN	Tot
**ANT**															0
**LAV**	2		3												5
**CLA**	7	8				4									19
**FIA**	4	4	11			12		2							33
**SON**	1		6	2				1							10
**SPO**	3	8	15	10	20										56
**TAR**			2			5									68
**FLY**		31	33	19	11	45				8	2	2	1		18
**CIC**		1	11	5	5	5		4			3				17
**RUG**	3	11	14	5	16	10		8	1						48
**MAC**		2	2	1		8		3	1	1					152
**STE**			10	1		3		2			1				34
**WHI**		4	13	9	1	12				6	2	1			7
**NAN**		2	3	1		3	1	4		2	3	2	2		23
**Tot**	20	71	123	53	53	107	17	11	5	3	24	2	1	0	490

**Table 4:** Ranks orders established before and after neutering in the absence of any sources of competition. We compared the rank order between all cats reported in bold. Kittens and newborns were not involved.

Rank Orders in the Absence of Any Sources of Competition	
Before Neutering	After Neutering
LEO	Dispersed before the 2nd period
**ANT**	**ANT**
**SON**	**LAV**
**LAV**	**SON**
**PIC**	**PIC**
PAL	Died before the 2nd period
PEL	Dispersed before the 2nd period
**RIG**	**RIG**
GNO	Died before the 2nd period
RED	Died before the 2nd period
**CLA**	**CLA**
**FIA**	**FIA**
SIL	Died before the 2nd period
kitten	**RUG**
newborn	**FLY**

**Table 5:** Ranks orders established before and after neutering in the presence of food.

Rank Orders in the Presence of Food	
Before Neutering	After Neutering
**ANT**	**ANT**
**LAV**	**LAV**
LEO	Dispersed before the 2nd period
PIC	Adopted before the 2nd period
**CLA**	**CLA**
PEL	Dispersed before the 2nd period
**FIA**	**FIA**
**SON**	**SON**
RIG	Dispersed before the 2nd period
RED	Died before the 2nd period
CAL	Died before the 2nd period
NER	Died before the 2nd period
Kitten	**SPO**
Newborn	TAR
Newborn	FLY
Newborn	CIC
Kitten	**RUG**
Newborn	MAC
Newborn	STE
Newborn	WHI
Newborn	NAN

We compared the rank order between all cats reported in bold.

## Supplementary Materials

The following are available online at https://www.mdpi.com/2076-2615/9/12/1105/s1, Table S1, Comparison matrices of submission without food. Table S2, Comparison matrices of submission with food.
